# Multi-Label Conditioned Diffusion for Cardiac MR Image Augmentation and Segmentation

**DOI:** 10.3390/bioengineering12080812

**Published:** 2025-07-28

**Authors:** Jianyang Li, Xin Ma, Yonghong Shi

**Affiliations:** 1 Academy of Engineering & Technology, Fudan University, Shanghai 200433, China; jianyangli20@fudan.edu.cn; 2 Digital Medical Research Center, School of Basic Medical Science, Fudan University, Shanghai 200032, China; 3 Shanghai Key Laboratory of Medical Image Computing and Computer Assisted Intervention, Shanghai 200032, China

**Keywords:** cardiac MRI segmentation, condition-guided diffusion model, data augmentation

## Abstract

Accurate segmentation of cardiac MR images using deep neural networks is crucial for cardiac disease diagnosis and treatment planning, as it provides quantitative insights into heart anatomy and function. However, achieving high segmentation accuracy relies heavily on extensive, precisely annotated datasets, which are costly and time-consuming to obtain. This study addresses this challenge by proposing a novel data augmentation framework based on a condition-guided diffusion generative model, controlled by multiple cardiac labels. The framework aims to expand annotated cardiac MR datasets and significantly improve the performance of downstream cardiac segmentation tasks. The proposed generative data augmentation framework operates in two stages. First, a Label Diffusion Module is trained to unconditionally generate realistic multi-category spatial masks (encompassing regions such as the left ventricle, interventricular septum, and right ventricle) conforming to anatomical prior probabilities derived from noise. Second, cardiac MR images are generated conditioned on these semantic masks, ensuring a precise one-to-one mapping between synthetic labels and images through the integration of a spatially-adaptive normalization (SPADE) module for structural constraint during conditional model training. The effectiveness of this augmentation strategy is demonstrated using the U-Net model for segmentation on the enhanced 2D cardiac image dataset derived from the M&M Challenge. Results indicate that the proposed method effectively increases dataset sample numbers and significantly improves cardiac segmentation accuracy, achieving a 5% to 10% higher Dice Similarity Coefficient (DSC) compared to traditional data augmentation methods. Experiments further reveal a strong correlation between image generation quality and augmentation effectiveness. This framework offers a robust solution for data scarcity in cardiac image analysis, directly benefiting clinical applications.

## 1. Introduction

Cardiovascular diseases are one of the major health issues worldwide, significantly impacting human’s quality of life [1]. Cine cardiac MRI captures dynamic images of the heart at different stages, providing detailed information about the cardiac structures and situations [2]. It is an important non-invasive diagnostic tool for cardiovascular diseases. Accurate segmentation of cardiovascular magnetic resonance (CMR) images is an important prerequisite in clinical practice to reliably diagnose and assess a number of major cardiovascular diseases. The segmentation of different regions of the heart, such as the ventricles and myocardium, helps physicians accurately assess the cardiac function (e.g., pumping capacity) and formulate personalized treatment plans.

In recent years, segmentation algorithms for cardiac MR images based on deep neural networks (DNNs) have demonstrated remarkable accuracy, establishing state-of-the-art performance [3]. The U-Net [4] segmentation network, as a validated effective DNN, has been extensively utilized in the algorithm models for cardiac MR image segmentation. The authors of [5] developed a fully automatic three-dimensional model based on the 3D U-Net network. The authors of [6] employed the U-Net network to segment three cardiac structures in the short-axis cine sequence and then used an ensemble classifier for the classification and diagnosis of cardiac diseases. The authors of [7] combined the interpretability of the level set method with the U-Net network’s ability to handle more complex images, proposing an automatic medical image segmentation method that integrates constraint terms and the level set method. These methods all leverage the segmentation capabilities of the U-Net network itself and propose new loss functions or combinable feature modules to enhance the model’s segmentation accuracy. However, when the dataset is small and limited, these DNN-based methods may lead to a severe overfitting phenomenon. The difficulty of collecting a large number of annotated medical samples has always been an objective issue in artificial intelligence medical imaging research. Therefore, data augmentation methods are a necessary solution to alleviate the limitation of having few annotated samples in medical datasets.

Data augmentation is a commonly used strategy in machine learning and deep learning to increase the variability and quantity of samples in an existing dataset [8]. This is achieved by applying a series of transformations to the original data or by creating new samples through generative models, without the need to collect additional samples. Data augmentation aims to enhance the diversity and variability of input data without increasing the volume of collected data, while promoting the deep neural network’s learning of invariance to transformed input data, enabling the network to generalize better to unseen data and improve its performance in new scenarios [9]. Therefore, in medical image research, data augmentation can effectively alleviate the issues of poor heterogeneity, inconspicuous features, and difficulties in annotation caused by the small scale of medical datasets. It also mitigates the overfitting and poor generalization problems in deep neural networks caused by the aforementioned issues [10,11].

The mainstream methods of data augmentation can generally be divided into two major categories: Transformation of original data and generation of artificial data [8]. The former typically increases the diversity of the dataset through various geometric transformations or pixel-level transformations. The latter, overcoming the limitations of image enhancement techniques in the former, can produce more diverse and challenging samples. Among them, Generative Adversarial Networks [12,13], Variational Autoencoders [14], and Diffusion Models [15] have all been applied in data augmentation methods for generating artificial samples. Specifically, due to the theoretical foundation of the diffusion model and its impressive ideal characteristics in terms of feature distribution coverage, ease of training, and scalability, they have quickly been applied to various visual tasks. Diffusion models do not require adversarial training and excel in the diversity of generated images compared to GANs, making them naturally more suitable for data augmentation. Diffusion models have achieved remarkable effects in natural image tasks. At the same time, diffusion models can also be constrained by prior conditions, enabling them to generate samples of specified types. The input conditions during data generation by diffusion models support multimodality, which can be categorized by text features, encoding features, segmentation maps, and more implicit feature vectors. In segmentation tasks, the most relevant to model performance is the segmentation map of the sample data, and this experiment will primarily utilize segmentation maps as input conditions.

Therefore, this paper proposes a conditional diffusion model image enhancement framework based on multi-label spatial mask constraints for cardiac applications. This framework leverages a conditional generative model to simulate the cardiac morphologies of different patients by generating pseudo-labels based on the spatial structural probability distribution of segmentation maps, thereby enhancing the diversity of training samples. The framework is mainly divided into three steps: First, a multi-pseudo label mask diffusion model that conforms to the spatial probability distribution is trained on a labeled cardiac MR dataset. Second, a cardiac MR image implicit diffusion model is trained using samples and mask pairs from the labeled dataset, with the mask serving as a conditional factor for generation. To ensure that the mask conditions are pixel-level aligned with the cardiac MR images, this paper integrates the SPADE module into the decoding process of the diffusion model, thus constraining the image generation. During image enhancement, the trained pseudo-label diffusion model is first used to generate multi-class cardiac masks, which serve as constraint conditions to control the generation of 2D cardiac pseudo-samples by the cardiac sample diffusion model. In the label generation process, due to the propensity of the diffusion model to generate scattered label points, a label screening strategy is designed to filter the generated labels, preventing the creation of noisy images during the cardiac image generation phase.

Our contributions are as follows:

- A diffusion-based data augmentation framework capable of generating cardiac MR images and their segmentation labels from scratch
- A multi-category cardiac mask model and a conditional cardiac MR image synthesis model
- Experiments demonstrate that our method achieves performance improvements on multiple datasets compared to traditional data augmentation methods.

## 2. Related Works

### 2.1. Traditional Data Augmentation Methods

Traditional data augmentation (DA) techniques aim to expand the diversity and size of training datasets by applying various transformations to existing samples. These methods primarily operate by introducing controlled variations that mimic real-world data characteristics, thereby enhancing the robustness and generalization capabilities of deep learning models [8]. Common traditional augmentation strategies encompass a variety of techniques, including geometric transformations, such as rotation, translation, scaling, horizontal or vertical flipping, and shearing, which are particularly effective in making models invariant to object pose and position. Photometric (color space) transformations, involving adjustments to brightness, contrast, saturation, and hue, are applied to simulate varying lighting conditions or sensor properties. Other methods include random erasing or occlusions, which simulate partial occlusions by applying masks or erasing parts of images, encouraging the model to learn features from incomplete information [16], and image mixing techniques, such as blending or cutting and pasting parts of images together, designed to create novel sample distributions, though often less conventional for direct visual interpretation. Among these, affine transformations and elastic transformations are particularly notable, especially in medical imaging; affine transformations are geometric mappings that preserve straight lines and parallelism, crucial for maintaining basic anatomical relationships while introducing variations in scale, rotation, and translation, while elastic transformations apply a spatial deformation field to images, allowing for the introduction of more complex, non-rigid local shape variations, which can be highly effective in simulating natural biological deformations of organs or lesions. While these pixel-level transformation techniques effectively improve the robustness of segmentation algorithms to variations seen within the training data distribution, they inherently generate highly correlated samples, which limits their capacity to genuinely enhance a network’s generalization ability to novel, unseen data populations beyond the initial training set. More recently, feature mixing methods, prominently represented by MIXUP [17], have emerged as a significant paradigm in deep learning data augmentation. MIXUP generates new training samples by linearly interpolating between two existing samples and their corresponding labels. From a human perspective, mixing images by averaging pixel values or performing cut-and-paste operations might appear counterintuitive or even generate visually meaningless examples [18], but nevertheless, these interpolation-based techniques have been empirically proven to significantly enhance the generalization ability of deep neural networks and improve their robustness against adversarial examples [19]. However, the application of generic MIXUP in medical image segmentation, especially concerning anatomical consistency, remains a subject of considerable debate [20], as the arbitrary mixing of pixel values or regions can easily corrupt the intricate and consistent anatomical structures crucial for accurate medical diagnoses, potentially leading to inconsistencies in segmented outputs and raising concerns for clinical interpretability and practice.

### 2.2. Generation of Artificial or Synthetic Samples

The generation of artificial or synthetic samples can yield more diverse and challenging samples, thereby overcoming the limitations of transformation-based image augmentation techniques. Generative Adversarial Networks (GANs) [12,13] are the most prevalent generative architecture in current research. GANs consist of two components: a generator and a discriminator. While the generator attempts to deceive the discriminator by presenting fake data as real, the discriminator strives to discern whether an image is genuine or synthetic. The success of GANs over Variational Autoencoders (VAEs) lies in their exceptional visual fidelity. Furthermore, with the advancements in GAN research, Conditional GANs (CGANs) and their subsequent study have gradually matured. CGANs can generate samples that better align with specific conditions set by researchers, which is crucial for medical image research, as disease analysis in medicine often involves images of particular diseases or scenarios. By utilizing conditional generative networks, data bias in datasets can be mitigated to a greater extent, and the conditional content can serve as pseudo-annotations for the generated data, acting as auxiliary information to support training in downstream tasks. However, employing GANs for image augmentation also confronts significant challenges. Notably, GANs inherently require training two subnetworks in an adversarial manner, which can easily lead to mode collapse [21]. This phenomenon occurs when the generator produces highly similar samples, limiting its generalization capability. Alternatively, if the discriminator is inadequately trained, it may produce hallucinations or obscure disease-indicative features, resulting in the generator producing a significant number of noisy images. This can deteriorate downstream training, leading to reduced performance in downstream models.

### 2.3. Diffusion Probabilistic Models

Diffusion Probabilistic Models (DPMs) have recently emerged as a leading force in image generation, consistently achieving state-of-the-art results [22,23,24,25,26]. A notable breakthrough in this field is the Denoising Diffusion Probabilistic Model (DDPM) [13], which leverages score matching and a learned Markov chain to progressively transform a Gaussian noise distribution into a desired target image distribution. This method has demonstrated superior performance in generating high-quality images.

Beyond basic image generation, DPMs have been extended to various image-to-image translation tasks. For example, Palette [26] introduces a versatile model that utilizes DMs by concatenating source and target images. This enables a wide array of applications, including inpainting, uncropping, image restoration, and colorization, all without requiring specific architectural modifications for each task. Palette consistently achieves high-fidelity results across these diverse applications.

Another significant advancement is the Latent Diffusion Model (LDM) [25], which optimizes the training process by separating it into two stages for improved efficiency. First, an autoencoder is trained to compress high-dimensional image data into a perceptually equivalent, lower-dimensional latent representation. Subsequently, the diffusion model itself is trained within this compact latent space. This approach significantly reduces computational complexity, enabling more efficient image generation and notably, allows for effective training on 3D images without the need for cumbersome patch cropping.

## 3. Methods

The goal of cardiac image segmentation model S is to learn a mapping from the space of cardiac images *X* to the space of pixel-level labels *Y*. Assuming the training set $D_{l}$ consists of *N* labeled data pairs, denoted as $D_{l}={\left\{\left(x_{i}^{l},y_{i}^{l}\right)\right\}}_{i=1}^{N}$, where $x_{i}^{l}\in R^{C\times W\times H}$ represents cardiac images, and $y_{i}^{l}\in {\left\{0,1,\ldots ,K-1\right\}}^{W\times H}$ represents segmentation maps, *K* being the number of classes (in this case, K=4, where 0 stands for background, 1 for the left ventricle, 2 for the left ventricular myocardium, and 3 for the right ventricle). In supervised learning, the objective function $L_{S}$ is used to quantify the probabilistic discrepancy between the predicted labels ${\hat{y}}_{i}$ and the ground truth labels $y_{i}^{l}$. The objective is to minimize this discrepancy with respect to a set of learnable parameters ω of the segmentation network, as shown in Equation (1):

(1) $$L=\min_{\omega }\sum_{i=1}^{N}L_{S}\left({\hat{y}}_{i},y_{i}^{l}\right)=\min_{\omega }\sum_{i=1}^{N}L_{S}\left(S\left(x_{i}^{l}\right),y_{i}^{l}\right)$$

After the application of data augmentation in supervised learning, the augmented dataset D is composed of the original labeled dataset $D_{l}$ and the augmented dataset $D_{a}$: $D=D_{l}\cup D_{a}$, where $D_{a}={\left\{\left(x_{i}^{a},y_{i}^{a}\right)\right\}}_{i=N+1}^{N+M}$, $x_{i}^{a}\in R^{C\times W\times H}$ represents the augmented cardiac images obtained through enhancement, and $y_{i}^{a}\in {\left\{0,1,\ldots ,K-1\right\}}^{W\times H}$ are the corresponding segmentation maps. Now, the objective function for training the segmentation network *S* can be computed through optimization as shown in Equation (2).

(2) $$L=\min_{\omega }\sum_{i=1}^{N}L_{S}\left(S\left(x_{i}^{l}\right),y_{i}^{l}\right)+\sum_{i=N+1}^{N+M}L_{S}\left(S\left(x_{i}^{a}\right),y_{i}^{a}\right)$$

Obviously, obtaining a rich and diverse set of reasonable pseudo samples, $x_{i}^{a}$, $y_{i}^{a}$, is crucial for enhancing the performance of cardiac region segmentation. Therefore, our goal is to generate virtual pseudo-labels $y_{i}^{a}$ using a diffusion model, given a limited number of training sample pairs $x_{i}^{l}$, $y_{i}^{l}$. Subsequently, based on these pseudo-labels, we aim to produce a certain number of reliable pseudo-images, $x_{i}^{a}$, to improve the model’s generalization capabilities and robustness.

**Figure 1:**
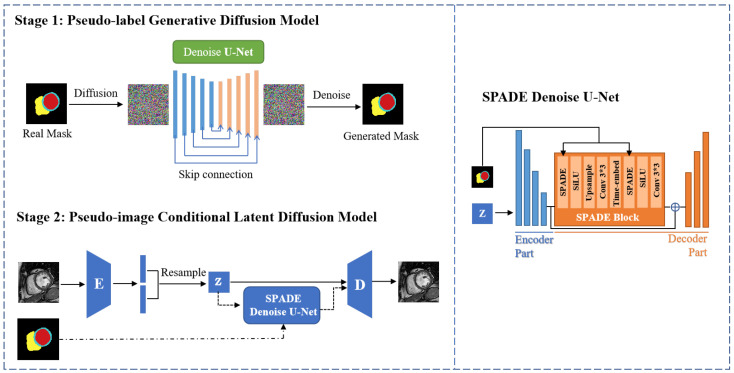
Enhanced framework for generating data through diffusion based on prior masks.

To achieve this goal, this paper proposes a data augmentation method based on conditional generation with cardiac multi-label spatial mask constraints, as shown in Figure 1. Initially, a pseudo-label generative diffusion model is trained based on the cardiac spatial structural mask, enabling the generation of pseudo-labels that conform to the spatial structural probability distribution within the label images. Subsequently, we propose a conditional generative latent diffusion network based on cardiac segmentation labels. The process starts by mapping cardiac images from the pixel space to an implicit feature space using an encoder, and then decoding to restore the training, with the aim of obtaining an effective and stable encoder-decoder and implicit feature space. Secondly, features within the implicit space are trained using a diffusion network. To ensure pixel-perfect alignment of input segmentation labels with the final generated images, a SPADE module is integrated into the decoding part of the diffusion model for image synthesis constraints. Furthermore, during the label generation process, this paper introduces a label screening strategy that helps the model filter out effective and reasonable label inputs into the subsequent conditional cardiac image generation model.

Next, we will introduce the pseudo-label generative diffusion model, pseudo-image generative latent diffusion model, and filtering strategy for effective labels.

### 3.1. Pseudo-Label Generative Diffusion Model

Cardiac mask labels can represent the structure of the heart; therefore, different mask labels can enhance the diversity of cardiac structures. The goal of this phase is to train a pseudo-label diffusion generative model $f_{DM}\left(\epsilon ,\theta \right)$, which takes random sample noise ϵ as inputs and generates pseudo-labels $y^{p}=f_{DM}\left(\epsilon ,\theta \right)$, resulting in a pseudo-label dataset $D_{plabel}={\left\{\left(y_{i}^{p}\right)\right\}}_{i=1}^{M}$, where M is the number of pseudo-labels. This process synthesizes a greater number of label images to be used as conditional labels in the second phase. The label images contain three spatial structural labels of the heart: the left ventricle, the left ventricle myocardium, and the right ventricle, allowing for direct learning of the basic cardiac spatial structure distribution information. This is referred to as the multi-structure label diffusion generative module, which can provide more direct ventricular structure information guidance for subsequent image generation.

**Figure 2:**
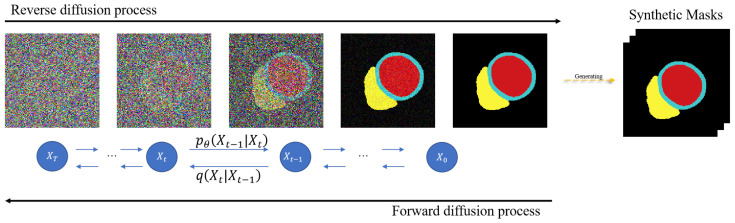
Generate pseudo labels based on the cardiac spatial structure.

For the sake of conciseness, the true cardiac mask structure is denoted as *y*, $y\in D^{l}$. To maximize data likelihood, the diffusion model defines both forward (also known as generative) and reverse processes, as shown in Figure 2. During the forward process, a small amount of Gaussian noise is sequentially added to the mask *y* over T steps, following the Equations (3) and (4):

(3) $$y_{t}=\sqrt{\alpha_{t}}y_{t-1}+\sqrt{1-\alpha_{t}}\epsilon_{t-1},\;t=1,\ldots ,T$$

(4) $$y_{t}=\sqrt{{\bar{a}}_{t}}y_{0}+\sqrt{1-{\bar{a}}_{t}}\epsilon_{t},\;t=1,\ldots ,T$$

where noise $\epsilon_{t}∼N\left(0,I\right)$, with I being the identity matrix. The set ${\left\{\alpha_{t}\in \left(0,1\right)\right\}}_{t=1}^{T}$ represents a schedule of variances, and ${\bar{a}}_{t}=\prod_{i=1}^{t}\alpha_{i}$. The result sequence $\left\{y_{0},\ldots ,y_{t}\right\}$ forms a Markov chain. Given $y_{t-1}$, the conditional probability of $y_{t}$ follows the Gaussian distribution in Equation (5):

(5) $$q\left(y_{t}|y_{t-1}\right)=N\left(y_{t};\sqrt{\alpha_{t}}y_{t-1},\left(1-\alpha_{t}\right)I\right)$$

In the reverse process, since $q(y_{t}|y_{t-1})$ is not easily estimated, a neural network model $p_{\theta }\left(y_{t-1}|y_{t}\right)$ is utilized to approximate $q(y_{t}|y_{t-1})$, which also follows the Gaussian distribution as described in Equation (6):

(6) $$p_{\theta }\left(y_{t-1}|y_{t}\right)=N\left(y_{t-1};\mu_{\theta }\left(y_{t},t\right),\Sigma_{\theta }\left(y_{t},t\right)\right)$$

The network needs to optimize the negative log-likelihood through the variational lower bound, as shown in Equations (7) and (8).

(7) $$\begin{array}{cc} -\log p_{\theta }\left(x_{0}\right) & \leq -\log p_{\theta }\left(x_{0}\right)+D_{\mathrm{KL}}\left(q\left(x_{1:T}|x_{0}\right)\|p_{\theta }\left(x_{1:T}|x_{0}\right)\right)\\ \; & =E_{q}\left[\log\frac{q(x_{1:T}|x_{0})}{p_{\theta }\left(x_{0:T}\right)}\right] \end{array}$$

(8) $$\begin{array}{cc} \; & L_{\mathrm{VLB}}=E_{q(x_{0:T})}\left[\log\frac{q(x_{1:T}|x_{0})}{p_{\theta }\left(x_{0:T}\right)}\right] \end{array}$$

The objective function is the variational lower bound loss: $L_{\mathrm{VLB}}=L_{T}+L_{T-1}+\ldots +L_{0},$ where each term except $L_{0}$ represents the Kullback–Leibler (KL) divergence between two Gaussian distributions. In practice, a simplified version of $L_{\mathrm{DM}}$ is commonly used [13], as shown in Equation (9):

(9) $$L_{DM}=E_{y\in D^{l},\epsilon_{t}∼N\left(0,I\right),t}\left[\right\|\epsilon_{t}-\epsilon_{\theta }\left(y_{t},t\right){\|}_{2}^{2}]$$

Once the network training is completed, denoising can be performed stepwise at random time points across the *T* time steps to generate new samples, as shown in Equation (10).

(10) $$y_{t-1}=\frac{1}{\sqrt{\alpha_{t}}}\left(y_{t}-\frac{1-\alpha_{t}}{\sqrt{1-{\bar{\alpha }}_{t}}}\epsilon_{\theta }\left(y_{t},t\right)\right)+\sigma_{t}z$$

where z∼N(0,I).

To synthesize cardiac mask structures, an unconditional Denoising Diffusion Probabilistic Model (DDPM) is trained on the original cardiac masks. Following [22], this unconditional DDPM adopts a U-Net architecture. Ultimately, this process generates a pseudo-label dataset $D_{\mathrm{plabel}}=\left\{y_{i}^{p}|i=1,\ldots ,M\right\}$.

### 3.2. Pseudo-Image Conditional Latent Diffusion Model

This stage involves synthesizing cardiac MR images conditioned on cardiac segmentation maps. In the absence of constraints, unconditional diffusion models will generate a diverse range of samples. There are generally two approaches to conditional synthesis of constrained images: classifier-guided diffusion [24] and classifier-free guidance [23]. Since classifier-guided diffusion requires training a separate classifier, which is not suitable for this task and incurs additional training costs, we opt for classifier-free guidance to control the sampling process. Additionally, because medical images are often high-resolution, and the MR image space is more complex compared to the label space, and diffusion models have substantial computational requirements for high-resolution images, to reduce the significant demand for computing time and resources, an implicit diffusion model [25] is trained. An autoencoder learns a space that is perceptually equivalent to the image space, significantly reducing computational complexity, after which the diffusion model learns the internal implicit space. This approach helps to enhance computational efficiency, and the U-Net architecture continues to effectively learn spatially structured data.

As shown in Figure 1, from the labeled dataset $D_{l}={\left\{\left(x_{i}^{l},y_{i}^{l}\right)\right\}}_{i=1}^{N}$, any image $x\in R^{C\times H\times W}$ is selected and encoded by the encoder *E* into the latent features z=E(x). The decoder *D* then reconstructs the image from the latent space, $\tilde{x}=D\left(z\right)=D\left(E\left(x\right)\right)$. Here, $z\in R^{c\times h\times w}$, and the encoder downsamples the image by a factor $f=\frac{H}{h}=\frac{W}{w}$, where *f* is a hyperparameter. Once a stable and effective autoencoder is trained, each image *x* can be encoded into its corresponding latent feature space. Subsequently, a diffusion network can be used to learn within the latent space, akin to Equation (11), to obtain the loss function in the latent space at this time:

(11) $$L_{LDM}=E_{z,\epsilon_{t}∼N\left(0,I\right),t}[∥\epsilon_{t}-\epsilon_{\theta }\left(z_{t},t\right){∥}_{2}^{2}]$$

Given that the forward process is fixed, during training, the latent features $z_{t}$ can be efficiently obtained from *E*, and the features *z* can be decoded into the image space with a single pass through *D*.

Cardiac labels and cardiac MR images possess distinct feature spaces. Simply connecting them within a denoising U-Net network or passing them through a cross-attention module can reduce image fidelity and result in unclear correspondence structures between synthesized cardiac MR images and their segmentation labels. Therefore, we consider employing a Spatially-Adaptive Normalization (SPADE) [27] module to correspond label information with cardiac images. During the decoding process of the conditional synthesis U-Net generative network, SPADE is constructed, and we include SPADE modules at different resolution layers of the network to leverage the multi-scale information of cardiac label structures. The encoder consists of stacks of residual blocks (Resblocks) and attention blocks (AttnBlocks). The decoder is a stack of SPADE Blocks and attention blocks. Each SPADE Block is composed of SPADE, SiLU, and Convolution, which takes feature maps and cardiac tags as inputs.

The SPADE module has been proven effective in semantic image synthesis by adjusting the normalized feature maps using spatially adaptive transformations learned from input semantic layouts, allowing for better preservation of semantic information compared to conventional normalization layers. This approach is particularly suitable for tasks such as semantic image synthesis, where the generation of realistic images from semantic masks is desired. By incorporating the SPADE module, the network can effectively propagate semantic information throughout the generative process, leading to the synthesis of images that are not only realistic but also aligned with the input semantic structures.

### 3.3. Filtering Strategy for Effective Labels

The pseudo-label generative diffusion model enables denoising the random Gaussian noise ϵ to generate pseudo-labels. However, the structure of these pseudo-labels influences the subsequent phase of cardiac MR image generation. We have identified four abnormal conditions that can occur after pseudo-label generation, which are crucial for the next phase:

(1) Disjointed parts within the cardiac structure; (2) Spatial configurations that do not align with clinical logic; (3) Excessively small pixel occupation by cardiac labels relative to the whole content; and (4) A large number of discrete labels appearing in the background.

In subsequent ablation studies, we evaluated the impact of including or excluding these abnormal labels. We discovered that the input of these abnormal labels affects the generation of pseudo-cardiac images and the performance of downstream segmentation tasks. It is essential to address these issues to enhance the quality and accuracy of the generated images and the subsequent diagnostic or therapeutic applications in the medical field.

## 4. Experiments

### 4.1. Cardiac MR Datasets

The heart dataset is derived from the M&Ms Challenge [28]. This public dataset primarily includes multi-disease cardiac MR data from various centers and different devices. We divided it into multiple sub-datasets according to the equipment and conducted experimental verification on the Canon and Siemens datasets. The Canon dataset includes 50 cases of cardiac MR data from patients, which we divided into a training set of 35 cases, a validation set of 5 cases, and a test set of 10 cases in a 7:1:2 ratio. The Siemens dataset includes data from 94 patients, which we also divided into a training set of 66 cases, a validation set of 9 cases, and a test set of 19 cases in a 7:1:2 ratio. Each case includes the left ventricle, left ventricular myocardium, and right ventricle in the end-diastolic and end-systolic frames, manually labeled by experts. We preprocessed the images, and to save computational costs, all images were centrally cropped and resized to 128×128.

### 4.2. Experiments Details

To validate the effectiveness of the proposed augmentation method, its performance was rigorously evaluated through a downstream image segmentation network. The experimental setup involved specific configurations for the image generation module and the segmentation task.

Within the image generation module, the pseudo-label generative diffusion model’s encoder and decoder each consist of six layers, with channel dimensions progressively increasing through 64, 128, 256, 512, up to 1024. Each layer within both the encoder and decoder comprises two ResNetBlocks, with the final three layers additionally incorporating Attention Blocks. This network was trained using the Adam optimizer with a learning rate of $10^{-4}$ and a batch size of 16. For the pseudo-image conditional latent diffusion model, the autoencoder component’s encoder and decoder each feature five layers, with channel dimensions progressing through 64, 128, 256, up to 1024. Each layer in both the encoder and decoder includes two ResBlocks, and Group Normalization is applied with 64 groups. This autoencoder network was trained using the Adam optimizer, with varying learning rates applied to different subsets of the training data. For the downstream image segmentation task, a U-Net architecture was employed, with both its encoder and decoder comprising four layers. This segmentation network was trained using the Adam optimizer with a learning rate of 0.0001 and a weight decay of 0.00001. The training epoch of the pseudo-label generative diffusion model is 1000 and the training epoch of the pseudo-image conditional latent diffusion model’s autoencoder network and ldm network is 500. The training epoch of the segmentation model is 400. All experiments in this study were conducted on a GTX A40 GPU.

### 4.3. Performance Evaluation of Methods

The experiment includes data augmentation and image segmentation. In the data augmentation phase, six different techniques are applied to increase the diversity and quantity of the training dataset, which is crucial for improving the robustness and generalization capability of the segmentation models. Each method has its unique way of altering the image data:

- No Data Augmentation: This serves as the baseline, where the original dataset is used without any augmentation.
- Affine Transformations: These include operations like rotation, translation, scaling, and shearing, which preserve the collinearity of points but not necessarily the distances.
- Elastic Deformations: Also known as non-linear transformations, these allow for more complex distortions that can simulate various deformations in the image.
- Pixel Intensity Transformations: Adjustments to the brightness, contrast, and color properties of the pixels to enhance the visibility and quality of the images.
- CutMix Method: A data augmentation technique that combines two images by cutting a portion from one image and pasting it into another, which encourages the model to learn from the context of mixed images.
- Proposed Method: The novel data augmentation approach introduced in this study, which aims to generate more realistic and diverse samples to improve segmentation performance.

The effectiveness of these methods is evaluated in the image segmentation phase, where the U-Net network, a popular choice for medical image segmentation due to its encoder-decoder structure with skip connections, is employed. The performance of the segmentation models is quantitatively assessed using the DSC coefficient, which measures the overlap between the predicted and actual segmentation masks, and the IoU value, which calculates the ratio of the overlapping area to the total area covered by the predicted and actual masks.

By comparing the DSC and IoU values across different augmentation methods, we can determine which approach contributes the most to the improvement of segmentation accuracy and model generalizability. This comprehensive experimental setup ensures a thorough evaluation of the proposed data augmentation method against existing techniques in the context of cardiac MR image segmentation.

## 5. Results and Discussion

Table 1 and Table 2 display the quantitative outcomes of employing various augmentation techniques on two separate datasets. The numerical values reported are the mean Dice coefficients for each technique. It is evident that the proposed method yields significant enhancements compared to other augmentation strategies, with the optimal and suboptimal methods highlighted in red and blue, respectively. Notably, the absence of any data augmentation results in the poorest performance of the segmentation model. Data augmentation through affine transformations significantly boosts the model’s performance. The incorporation of random elastic transformations further augments accuracy. However, augmentation that solely alters the pixel intensity of images can lead to a decline in segmentation model performance. The CutMix method also ameliorates the segmentation model’s effectiveness. Despite the counterintuitive appearance of images generated by CutMix, preserving the authentic anatomical forms may not be essential for neural networks to attain superior segmentation outcomes. On the Canon dataset, our method achieved an average DSC improvement of 0.19 over the baseline model without augmentation, with increases of 0.16 for the Left Ventricle (LV), 0.23 for the Myocardium (MYO), and 0.16 for the Right Ventricle (RV). Regarding the IoU values, an average enhancement of 0.18 was observed, with improvements of 0.16 for the LV, 0.24 for the MYO, and 0.15 for the RV.

**Figure 3:**
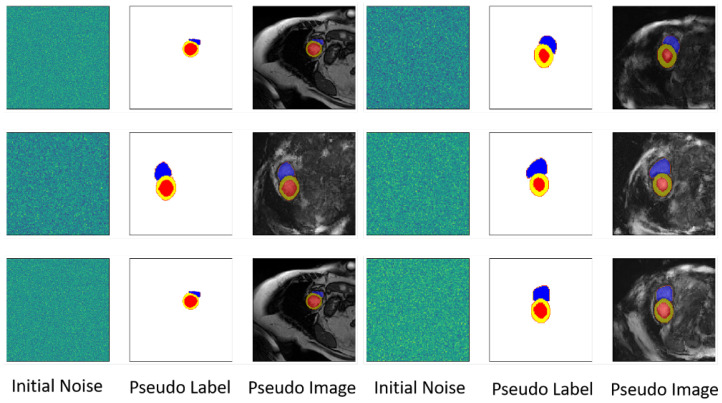
Illustration of the proposed framework’s generative process for cardiac MRI images. The left panel displays the initial noise input. The middle panel shows the generated pseudo-label map, which guides the image synthesis. The right panel presents the final pseudo-image, generated based on the pseudo-label, demonstrating the framework’s ability to produce synthetic cardiac MRI scans.

**Figure 4:**
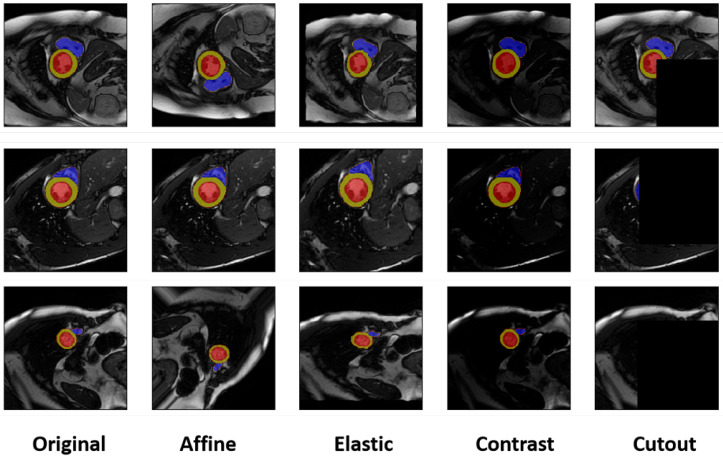
Visualizations of comparative data augmentation methods applied to cardiac MRI images.

In contrast to other data augmentation techniques, our method uniquely encapsulates the viability of preceding approaches on two crucial fronts. Firstly, by emulating the state of the label space, the framework is capable of generating pseudo-label maps that adhere stochastically to the distribution of the original label space. This inherent characteristic ensures significant enhancements to the structural aspects of the image space, mirroring and even extending the benefits typically achieved through conventional geometric transformations like elastic or affine transformations. Secondly, the subsequent generative training of cardiac MR images ensures robust variability in the intensity at each pixel point, thereby substantially augmenting the overall diversity of the images. Consequently, the two distinct phases we propose correspond precisely to the spatial variations in the image and the alterations in pixel intensity that are characteristic of conventional data augmentation methods. This dual-phase approach allows our framework to synthesize realistic and diverse data while maintaining anatomical plausibility, which is often a challenge for simpler augmentation techniques. Figure 3 illustrates the generation process of our proposed framework, while Figure 4 presents the visualization results of other comparative data augmentation methods, offering a clear visual comparison of the distinct outputs.

## 6. Discussion

Our proposed framework demonstrates significant potential for broader application beyond the scope of this study, opening avenues for future research and deployment in diverse medical imaging contexts.

Firstly, the adaptability of our method is highlighted by its extensibility to segment other anatomical regions. By simply adjusting the training data to include images of different organs, such as the liver, brain, or kidneys, along with their corresponding mask conditions, the framework can be retrained to accurately delineate these new structures. This modularity suggests that the underlying principles of our approach are not confined to a single anatomical area but can be generalized across various body parts, making it a versatile tool for comprehensive medical image analysis.

Secondly, the framework’s applicability extends to different imaging modalities. While this study primarily focused on Cardiac MRI, our diffusion model’s core design allows for its retraining on the unique characteristics of other image types, such as CT or ultrasound, and their associated labels. This flexibility means the model can learn to interpret and generate segmentations from distinct data representations, greatly expanding its utility in clinical practice where multiple imaging techniques are often used in conjunction.

However, transitioning to these new domains or modalities does come with potential challenges and considerations. Variations in image quality, resolution, and pathological appearance across different anatomical regions or imaging modalities can significantly impact model performance. For instance, the inherent noise levels in ultrasound, the anisotropic resolution in some CT scans, or the diverse manifestations of diseases in different organs (e.g., tumors in liver vs. brain) would necessitate careful data curation and potentially modality-specific architectural adaptations or training strategies. Addressing these nuances will be crucial for ensuring robust and accurate segmentation performance in expanded applications.

## 7. Conclusions

Deep learning approaches have found success in medical image analysis, with one key requirement being access to extensive datasets with annotations. In the study, we introduced a data augmentation method leveraging a conditional diffusion model constrained by multi-label structures of the heart. This method was designed to tackle the scarcity of data in training datasets comprising 2D cardiac images. Our approach was realized through three key contributions: (1) utilizing a diffusion model to understand the spatial relationships among different cardiac multi-label structures; (2) generating cardiac MR images under the constraints of these multi-label structures; and (3) mitigating the generation and input of detrimental pseudo-samples by filtering out anomalous labels. In our experiments, we observed a propensity for the emergence of anomalous images during both phases of image generation and noted that the inference time for a single model was considerably long. To address this, we opted for the network architecture of LDM during the image generation phase to minimize computational expenditure. Furthermore, in the inference phase, we employed the DDIM computational strategy to accelerate the inference process, reducing the steps from an original 1000 to 50, thereby achieving an 80% reduction in time. The fidelity of image generation is intricately linked to the outcomes of the segmentation network; higher similarity in generated images correlates with superior performance enhancements.

## Figures and Tables

**Table 1:** Evaluation of different augmentation methods using DSC and IoU metrics in the Siemens dataset. The red characters represent the optimal enhancement method.

Method	Dice				IoU			
	Mean Dice	LV	MYO	RV	Mean IoU	LV	MYO	RV
UNET-no aug	0.8039	0.828	0.7675	0.8163	0.7255	0.7975	0.6053	0.7737
UNET-Affine aug	0.8188	0.8468	0.7865	0.8231	0.7503	0.8396	0.6338	0.7776
UNET-Elastic Aug	0.8244	0.8740	0.7803	0.8191	0.7281	0.7960	0.6152	0.7733
UNET-Contrast Aug	0.5515	0.5622	0.4918	0.5878	0.5214	0.5342	0.4474	0.5681
UNET-CutMix Aug	0.7967	0.8511	0.7605	0.7877	0.7460	0.8135	0.6895	0.7448
UNET-Ours	0.8291	0.8763	0.7888	0.8290	0.7755	0.7151	0.6589	0.7802

**Table 2:** Evaluation of different enhancement methods using DSC and IoU metrics in the Canon dataset. The red characters represent the optimal enhancement method.

Method	Dice				IoU			
	Mean Dice	LV	MYO	RV	Mean IoU	LV	MYO	RV
UNET-no aug	0.6644	0.7226	0.5823	0.6883	0.6129	0.6865	0.5096	0.6433
UNET-Affine aug	0.7606	0.7775	0.8136	0.6907	0.7093	0.7451	0.7361	0.6467
UNET-Elastic Aug	0.7331	0.8333	0.6504	0.7157	0.6749	0.7940	0.5740	0.6567
UNET-Contrast Aug	0.5916	0.5987	0.5249	0.6443	0.5622	0.5753	0.4848	0.6200
UNET-CutMix Aug	0.7999	0.8503	0.7567	0.7943	0.7504	0.8173	0.6854	0.7509
UNET-Ours	0.8470	0.8841	0.8155	0.8416	0.7961	0.8488	0.7441	0.7954

## Data Availability

No new data were created or analyzed in this study.
